# Evolving precision: Updates in targeted therapy for cholangiocarcinoma

**DOI:** 10.1097/HEP.0000000000001541

**Published:** 2025-09-22

**Authors:** Giulia Tesini, Halima Ibrahim, Lorenza Rimassa, Chiara Braconi

**Affiliations:** 1 School of Cancer Sciences, University of Glasgow, Glasgow, UK; 2 Department of Biomedical Sciences, Humanitas University, Pieve Emanuele, Milan, Italy; 3 Beatson West of Scotland Cancer Centre, Glasgow, UK; 4 Medical Oncology and Hematology Unit, Humanitas Cancer Center, IRCCS Humanitas Research Hospital, Rozzano, Milan, Italy; 5 CRUK Scotland Cancer Centre, Glasgow, UK

**Keywords:** biliary tract cancer, drug resistance, molecular diagnostic techniques, molecular targeted therapy, new actionable targets

## Abstract

The development of Next-Generation Sequencing (NGS) techniques for extended genomic profiling has led to the identification of actionable molecular alterations in approximately half of the patients with biliary tract cancer (BTC), with the highest incidences among those with intrahepatic cholangiocarcinoma. Targeted drugs have demonstrated the ability to confer clinical benefit while maintaining a manageable safety profile. As a result, despite the lack of a head-to-head comparison with standard second-line chemotherapy, they are now recommended for patients with advanced disease who are still fit after progression to first-line palliative systemic anti-cancer treatment. In this review, we will contextualize the results observed with targeted drugs in clinical trials within the framework of clinical practice. We will provide an overview of available single-gene analyses that should be considered in case of lack of access to NGS, defining testing priorities, differences in yields, and therapeutic implications. Lastly, we will discuss future perspectives in the field of precision medicine for BTC, focusing on new strategies to overcome treatment resistance, on the optimal collocation of targeted drugs in the treatment algorithm, and on newly identified actionable alterations for which compounds are currently under investigation.

## INTRODUCTION

Biliary tract cancers (BTCs) represent a diverse group of malignancies originating from the epithelial cells lining the biliary tree.^1^ These include cholangiocarcinoma (CCA), gallbladder cancer (GBC), and carcinoma of the ampulla of Vater. CCA is anatomically categorized into intrahepatic, perihilar, and distal subtypes.^1^ While BTCs remain relatively rare, their incidence continues to rise in regions where they have traditionally been more prevalent, such as Southeast Asia—particularly Thailand—China, South Korea, and Japan compared with the Western countries.^2,3^ This increase is likely attributed to factors such as exposure to environmental toxins and chronic infections.^2,4^

In 2021, there were 216,768 new cases of BTC globally, with an age-standardized incidence rate of 2.71 per 100,000 person-years, and the age-standardized death rate was 2.23 per 100,000 person-years.^4^ The burden of BTC was higher in individuals aged 75 and above, experiencing 5–10 times higher mortality rates compared with the general population.^4^ However, more recent data show how the incidence of BTC is rising in younger patients, with up to 30% of new cases occurring in people younger than 65, generating an impact on the social workforce of each country.^5,6^ Notably, a rising trend in incidence has been observed across Western countries, including the United States, the United Kingdom, and parts of Europe, with the greatest increase in incidence being observed among people aged 18–44 years.^7,8^ This trend is primarily driven by an increase in intrahepatic cases, influenced by factors such as a rise in conditions like chronic liver diseases and metabolic dysfunction–associated steatohepatitis, improved imaging technologies, and heightened public and medical awareness.^9^

Despite the advancements in diagnostic techniques, about 60%–70% of patients are diagnosed with advanced or metastatic disease, at which point curative interventions like surgery or liver transplantation are no longer viable. This is primarily due to the indolent and non-specific nature of early symptoms, which often go unnoticed until the disease has progressed to an advanced stage.^10^ In these cases, treatment generally shifts toward locoregional therapies and palliative systemic anti-cancer treatment (pSACT).

The standard first-line treatment for advanced BTC has historically been systemic chemotherapy, particularly the combination of gemcitabine and cisplatin as established by the ABC-02 trial.^11^ The TOPAZ-1 trial introduced the addition of the anti–programmed death ligand 1 agent durvalumab to gemcitabine and cisplatin, resulting in improvement in median overall survival (OS).^12–14^ Similarly, in the KEYNOTE-966 trial, combining the anti–programmed death-1 (PD-1) pembrolizumab to cisplatin and gemcitabine lead to an OS benefit.^15,16^ While chemoimmunotherapy regimens have offered modest gains in clinical outcomes, most patients eventually experience progressive disease (PD) after a median of 7–8 months, necessitating second-line options. For a long time, there was no consensus on how to treat BTC after first-line therapy failure, and decisions were made based on physician experience and individual patient condition rather than solid evidence, focusing more on improving or maintaining quality of life (QoL). This changed with the ABC-06 phase III trial, which compared modified FOLFOX [5-fluorouracil (5-FU), leucovorin, and oxaliplatin] to active symptom control in patients who had progressed after first-line gemcitabine and cisplatin. The study showed a statistically significant—albeit small—improvement in median OS (6.2 mo vs. 5.3 mo), establishing FOLFOX as a standard second-line treatment.^17^

Following ABC-06, the NIFTY and NALIRICC trials provided further insight into potential second-line therapies. The NIFTY trial, conducted in South Korea, evaluated liposomal irinotecan (nal-IRI) in combination with 5-FU and leucovorin. It showed an improvement both in objective response rate (ORR) (12.5% vs. 3.5%) and in survival outcomes [progression-free survival (PFS): 4.2 vs. 1.7 mo; OS: 8.6 vs. 5.3 mo] compared with 5-FU and leucovorin alone, with manageable side effects.^18^ In contrast, the German NALIRICC study tested a similar regimen but found inconsistent results, confirming the increased ORR with nal-IRI, but not the survival benefits (PFS: 2.75 mo vs. 2.3 mo; OS: 6.9 mo vs. 8.21 mo), despite higher toxicity.^19^ The benefit in ORR and PFS with doublet chemotherapy, as well as the association between ethnicity and incidence of adverse events (AEs), has been confirmed in a pooled analysis of the 2 trials.^20^

Even though these chemotherapy regimens have now entered routine clinical practice, second-line pSACT continues to face major limitations. Only 40% of patients are able to receive further therapy after first line, often due to rapid clinical deterioration, poor performance status, or coexisting illnesses.^5,21^ These findings highlight the complexity of BTC treatment and underscore the need for the development of personalized therapeutic approaches that can address the biological diversity of this type of cancer, with the potential to improve survival and maintain QoL, while exploring alternative strategies to expose patients to effective pSACT earlier in their management.

A significant shift in BTC treatment has come with the advent of modern genomic profiling, as it has opened the door to precision-targeted treatments that are more tailored and potentially more effective. Approximately half of BTCs harbor actionable targets, with variable incidences based on anatomical subtype and peaking in intrahepatic CCA.^21^ The most common drivers in BTC are *fibroblast growth factor receptor 2* (*FGFR2*) fusions and rearrangements (f/r), *isocitrate dehydrogenase 1* (*IDH1*) R132 mutations, human epidermal growth factor receptor 2 (HER2) overexpression/*erb-b2 receptor tyrosine kinase 2* (*ERBB2*) amplifications, and *BRAF* V600E mutations. Less frequent alterations include *Kirsten rat sarcoma virus* (*KRAS*) G12C mutations, *neurotrophic tyrosine receptor kinase* (*NTRK*) fusions, and *rearranged during transfection* (*RET*) fusions.

This change has now moved beyond just clinical research: it is increasingly shaping real-world practice.^21^ Regulatory approvals of targeted therapies like pemigatinib and futibatinib for tumors with *FGFR2* f/r, and ivosidenib for *IDH1*-mutated BTCs, exemplify this progress.^22–24^ As molecular testing becomes more widespread, precision oncology is expected to redefine BTC treatment, bringing a shift from purely palliative care to more personalized and potentially transformative therapeutic strategies.

This review sets out to examine how precision oncology is changing the treatment landscape for BTC, with a focus on recent progress in molecular profiling, the clinical relevance of new targeted therapies, and their role in driving a shift toward more individualized treatment strategies. Even though cross-trial comparisons are inaccurate from a statistical perspective, for the purpose of this paper we will compare different studies with the goal of providing insight into how to navigate the growing pool of targeted agents available for patients with BTC.

## TARGETED THERAPIES

Targeted therapies represent the standard of care for patients with relevant molecular alterations who remain fit after PD to first-line pSACT.^22–24^ Notably, no head-to-head comparison between chemotherapy and targeted drugs has ever been performed in a phase II/III trial, as all studies on targeted agents were designed before FOLFOX entered the treatment algorithm for BTC in 2021. Besides, large randomized clinical trials in this setting are difficult to conduct, as they face challenges related to slow accrual. For this reason, the evidence in favor of the majority of targeted agents derives from phase II single-arm studies or from basket trials where few patients with BTC were included (Table 1). Ivosidenib is the only drug that was evaluated in a phase III randomized trial, but the control arm was placebo.^28,29^

**TABLE 1:** Overview of clinical trials on targeted therapies for patients with biliary tract cancer

Trial	Drug	Target	Phase of trial	Control arm	Patients with BTC (n)	Max n of prior lines of therapy	Route	Dose	Treatment schedule
ABC-06^17^	FOLFOX	n/a	III	ASC	162 (overall)	1	i.v.	Oxa 85 mg/m^2^, L-FA 175 mg (or FA 350 mg), 5-FU 400 mg/m^2^ (bolus), 5-FU 2400 mg/m^2^ as a 46-hour infusion	Q2W for 12 cycles
FIGHT-202^25,26^	Pemigatinib	*FGFR2* f/r	II	n/a	108	More than 3	Oral	13.5 mg	OD 2 wk on/1 wk off →
FOENIX–CCA2^27^	Futibatinib	*FGFR2* f/r	II	n/a	103	More than 3	Oral	20 mg	OD in 21-day cycles →
ClarIDHy^28,29^	Ivosidenib	*IDH1* R132	III	Placebo	187 (overall)	2	Oral	500 mg	OD in 28-day cycles →
HERIZON–BTC-01^30,31^	Zanidatamab	*HER2* amplification/overexpression	IIb	n/a	87	2	i.v.	20 mg/kg	Day 1, 15 of 28-day cycle →
DESTINY–PanTumor02^32^	T-DXd	*HER2* amplification/overexpression	II	n/a	41 (16 HER2 3+)	More than 5	i.v.	5.4 mg/kg	Q3W →
KCSG–HB19-14^33^	FOLFOX + trastuzumab	*HER2* amplification/overexpression	II	n/a	34 (all Korean)	2	i.v.	Trastuzumab: 6 mg/kg on C1, 4 mg/kg from C2; FOLFOX as per ABC-06 trial	Q2W →
MyPathway^34^	Pertuzumab + trastuzumab	*HER2* amplification/overexpression	IIa	n/a	39	2	i.v.	Pertuzumab: 840 mg on C1, then 420 mg; Trastuzumab 8 mg/kg on C1, then 6 mg/kg	Q3W →
KEYNOTE-158^35–37^	Pembrolizumab	MSI-H/MMRd	II	n/a	22	More than 5*	i.v.	200 mg	Q3W for 35 cycles
ROAR^38,39^	Dabrafenib + trametinib	*BRAF* V600E	II	n/a	43	NR	Oral	Dabrafenib 150 mg Trametinib 2 mg	Dabrafenib BID and trametinib OD in 28-day cycles →
NAVIGATE^40^***	Larotrectinib	*TRK* fusions	II	n/a	4	More than 3	Oral	100 mg	BID →
Lu et al., 2023^41,42^	Entrectinib	*NTRK* fusions	I/II	n/a	1	More than 2*	Oral	600 mg	OD →
LIBRETTO-001^43^	Selpercatinib	*RET* fusions	I/II	n/a	2	NR	Oral	160 mg	BID →
ARROW^44^	Pralsetinib	*RET* fusions	I/II	n/a	4	More than 3*	Oral	400 mg	OD →
KRYSTAL-1^45^	Adagrasib	*KRAS* G12C	II	n/a	12	5**	Oral	600 mg	BID →

*Overall cohort; **Biliary cohort; ***Gastrointestinal cohort; → until PD or unacceptable toxicity.

Abbreviations: 5-FU, 5-fluorouracil; ASC, active symptom control; BID, *bis in die* (twice in a day); C, cycle; f/r, fusion or rearrangement; FA, folinic acid; i.v., intravenous; MMRd, mismatch repair deficient; MSI, microsatellite instability high; n/a, not applicable; NR, not reported; OD, once daily; Oxa, oxaliplatin; PD, progressive disease; QnW, every n weeks.

Nonetheless, most targeted drugs have been approved by the Food and Drug Administration (FDA) and/or the European Medicines Agency (EMA), and are used in clinical practice where available. The reasons are various. First of all, it is notable that all targeted agents have yielded better outcomes than chemotherapy, especially considering that often they have been assessed in heavily pretreated patients (Table 1). Real-world studies confirm that targeted treatment provides a benefit in OS over chemotherapy. In a single-center retrospective cohort of 79 patients, those who received targeted therapies in second or third line had a 5-month improvement in median OS from diagnosis compared with those who received standard second-line pSACT; the OS benefit was maintained even when considering survival from the start of second-line treatment, with an absolute median difference of 4 months.^46^ More recently, in a large international multicenter cohort of over 900 patients, receiving matched therapy for an actionable target was found to be a strong independent favorable prognostic factor and led to a statistically significant improvement in median OS and 3-year OS rates among patients who received targeted therapy compared with those who did not.^47^ Of note, the level of evidence for the use of targeted treatment against a specific actionable alteration as measured according to the European Society of Medical Oncology Scale of Clinical Actionability for molecular Targets (ESCAT) seems to be relevant: patients with ESCAT I–II molecular alterations showed better clinical outcomes compared with those with ESCAT III–IV alterations across different retrospective cohorts, underscoring the importance of accurate target selection for the success of personalized treatment.^46,48^

Furthermore, targeted agents are more easily manageable, as they have an oral route of administration or can be administered from a peripheral vein, not requiring central venous catheter placement to allow for the 46-hour-long 5-FU infusion that is a part of FOLFOX (Table 1). Lastly, tolerability appears adequate, with rates of grade 3–4 treatment-related AEs (TRAEs) comparable—if not better—than the ones observed with chemotherapy (Table 2).

**TABLE 2:** Adverse events of second-line chemotherapy and targeted therapies in patients with biliary tract cancer

Trial	Drug	Any G TRAEs (%)	G3-4 TRAEs (%)	Most common any G TRAEs	Most common G3-4 TRAEs	TEAEs leading to		
						Interruption/ dose delay (%)	Dose reduction (%)	Discontinuation (%)
ABC-06 (n=81)^17^	FOLFOX	G1–2: 46 G3–5: 38	38	Neuropathy (64%), fatigue (58%), nausea (37%)	Neutropenia (12%), fatigue (11%), infection (10%)	NR	NR	12
FIGHT-202 (n=147)^25,26^	Pemigatinib	91.8	32.7	↑Phosphatemia (53.7%), alopecia (46.3%), diarrhea (36.1%)	↓Phosphatemia (8.8%), stomatitis (6.1%), arthralgia and PPES (4.1%)	42.2	13.6	10.2
FOENIX–CCA2 (n=103)^27^	Futibatinib	99	G3: 56 G4: 1	↑Phosphatemia (85%), alopecia (33%), dry mouth (30%)	↑Phosphatemia (30%), ↑AST (7%), stomatitis and fatigue (6%)	50	54	2
ClarIDHy (n=121)^28,29^	Ivosidenib	63	6	Diarrhea (21%), nausea (21%), fatigue (16%)	↓Phosphatemia (2%), fatigue (2%), anemia (1%)	NR	3	6
HERIZON–BTC-01 (n=87)^30,31^	Zanidatamab	72	21	Diarrhea (32%), IRR (32%), nausea (8%)	Diarrhea (5%), ↓LVEF (3%), anemia (3%)	6	3	2
DESTINY–PanTumor02 (n=41)^32^**	T-DXd	80.5	39	Nausea (46.3%), anemia (24.4%), diarrhea (19.5%)	NR	31.7	24.4	19.5
KCSG–HB19-04 (n=34)^33^	FOLFOX + trastuzumab	100	G3: 68 G4: 26	Neutropenia (62%), peripheral neuropathy (50%), anemia (24%)	Neutropenia (29%), anemia (15%), peripheral neuropathy (12%)	NR	88	0
MyPathway (n=39)^34^	Pertuzumab + trastuzumab	62	8	Diarrhea (26%), ↑ ALT (10%), or AST (10%), IRR (10%)	↑ALT, ↑AST, ↑ALP or bilirubin	18	3	0
KEYNOTE-158 (n=373)^35–37^*	Pembrolizumab	67	13	Fatigue (13.4%), pruritus (13%), diarrhea (12%)	↑ALT, diarrhea, fatigue, rash (all 1%)	NR	n/a	8.3
ROAR (n=43)^38,39^*	Dabrafenib + trametinib	87.9	NR	Pyrexia (40.8%), fatigue (25.7%), chills (25.7%)	NR	56.3	44.2	13.6
NAVIGATE (n=44)^40^***	Larotrectinib	71	16	↑ ALT (18%), ↑AST (16%), anemia, diarrhea, dizziness, ↓PLT (14% each)	↑AST or ALT (5%), nausea, anemia, and ↓PLT (2% each)	NR	NR	18
Su et al., 2023 (n=296)^41,42^*	Entrectinib	NR	NR	Dysgeusia (34.5%), constipation (29.1%), diarrhea, and ↑weight (28.7% each)	NR	34.1	24	6.4
LIBRETTO-001 (n=45)^43^*	Selpercatinib	G1–2: 51 G3: 38	38	Dry mouth (29%), ↑ALT (18%), ↑AST (18%)	↑ALT (16%), hypertension (13%), ↑AST (11%)	NR	31	2
ARROW (n=29)^44^*	Pralsetinib	86	69	↑AST (38%), ↑ALT (34%), neutropenia (34%)	Neutropenia (31%), anemia (14%), ↑AST (10%)	59	45	0
KRISTAL-1 (n=63)^45^*	Adagrasib	96.8	G3: 25.4 G4: 1.6	Nausea (49.2%), diarrhea (47.6%), fatigue (41.3%)	Fatigue (6.3%), QT prolongation (6.3%), ↑AST (3.2%)	44.4	39.7	0

*Overall cohort; **Biliary cohort; ***Gastrointestinal cohort; ↑increased; ↓ decreased.

Abbreviations: ALT, alanine aminotransferase; AST, aspartate aminotransferase; G, grade; IRR, infusion-related reaction; LVEF, left ventricular ejection fraction; n/a, not applicable; NR, not reported; PPES, palmar-plantar erythrodysesthesia syndrome; TEAE, treatment-emergent adverse event; TRAE, treatment-related adverse event.

### Response rate

**FIGURE 1:**
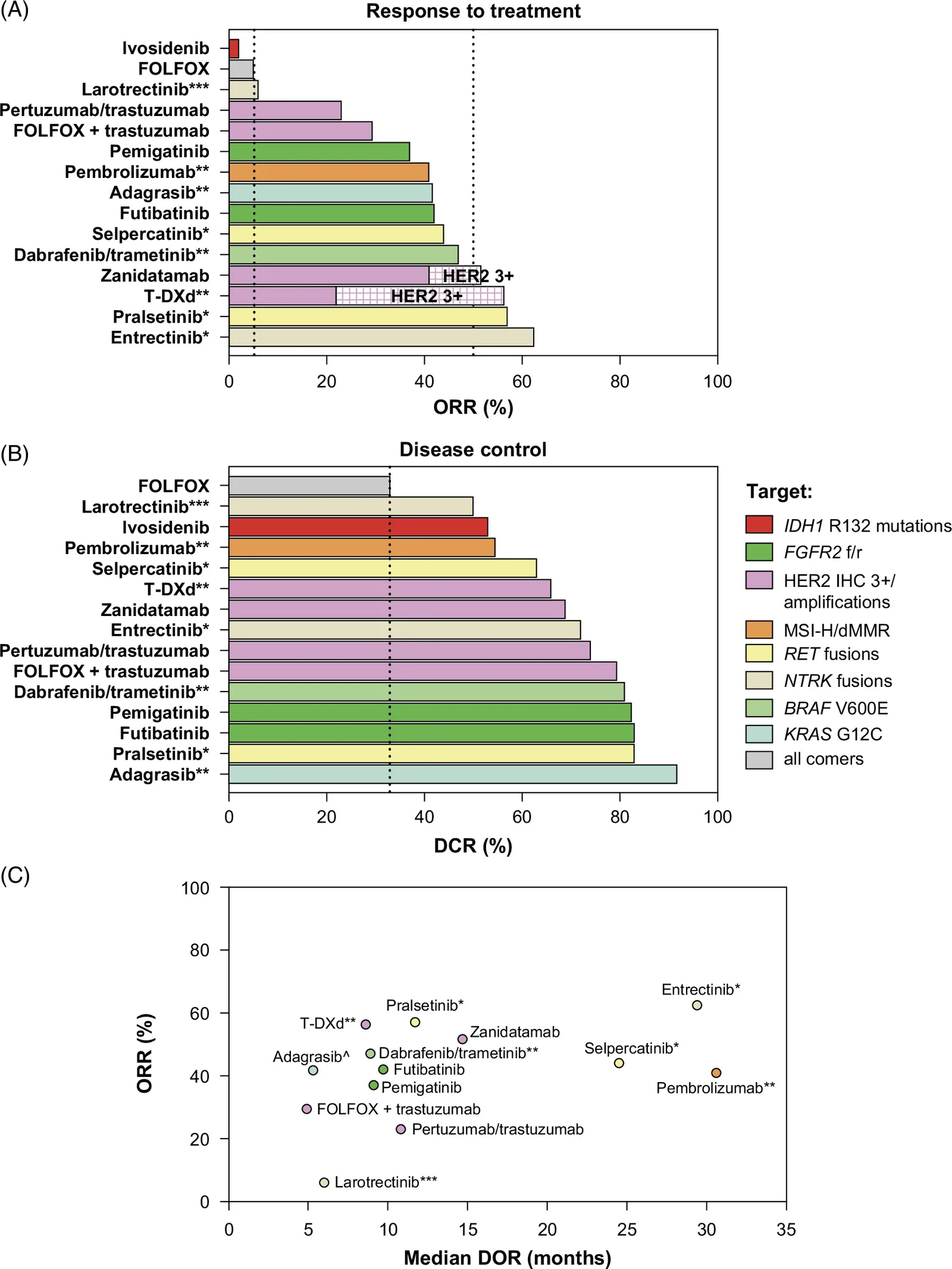
Treatment efficacy for patients with BTC receiving targeted therapy across key clinical trials compared to chemotherapy with FOLFOX.^17,25–45^ Panel (A) compares ORRs, while panel (B) compares DCRs. ORRs are plotted against their respective median DOR in panel (C). In panel C, for anti-HER2 agents, ORRs observed in patients with HER2 3+ tumors are plotted; data on median DOR for ivosidenib and FOLFOX are missing, and they are not included in this graph. Created with GraphPad and Adobe Illustrator. *Results relative to the overall cohort; **Results relative to the biliary cohort; ***Results relative to the gastrointestinal (non-colorectal) cohort. ^Data relative to ORR refers to the biliary cohort, while the median DOR is the one for the overall study cohort. Abbreviations: DCR, disease control rate; dMMR, deficit in mismatch repair proteins; DOR, duration of response; f/r, fusions/rearrangements; IHC, immunohistochemistry; MSI-H, microsatellite instability high; ORR, objective response rate; T-DXd, trastuzumab deruxtecan.

Achieving disease control during second-line pSACT aims at improving symptom control and delaying the onset of new cancer-related symptoms, thus guaranteeing a better QoL. FOLFOX demonstrated an objective response rate (ORR) of 5% and a disease control rate (DCR) of 33%.^17^ All targeted therapies have yielded better outcomes in this regard, with the exception of ivosidenib, which showed an ORR of 2%, but a DCR of 53% (Figures 1A, B).^28,29^ This is consistent with its mechanism of action. R132 mutations involving the active site of IDH1 determine an increase in the oncometabolite D-2-hydroxyglutarate (2-HG) within the cell, leading to altered cellular differentiation and histone hypermethylation.^49^ Ivosidenib can revert these changes, promoting hepatocyte differentiation and decreasing proliferation rate, thus acting as a cytostatic rather than a cytotoxic drug.^50^ For this reason, considering the benefit in long-term disease control and survival, as discussed later on, in patients with *IDH1*-mutated BTC, it may be sensible to switch to targeted treatment at the early signs of PD to first-line pSACT, before the onset of mass effect symptoms, which would not be controlled by ivosidenib due to its low ORR.

In general, the ORRs of the majority of targeted agents are sitting at 40% (Figure 1A). NTRK inhibitors showed even better outcomes. Entrectinib yielded responses across all tumor types included in the trial, with 16.5% of the patients achieving a complete response (CR); overall, the ORR was 62.4%, with consistent results even among patients with intracranial disease, who represent a population with poor prognosis. Only one patient with BTC was included, and a partial response (PR) was observed,^41,42^ making the case for the investigation of *NTRK* fusions in patients with BTC, despite their rarity.^51^ By contrast, larotrectinib, albeit effective in colorectal cancer (ORR: 44%), demonstrated worse outcomes in other gastrointestinal cancers, with an ORR of 6%; in particular, among 4 patients with BTC, 3 were eligible for radiological assessment, and none achieved a PR, but 2 had stable disease (SD).^40^ RET inhibitors yielded promising outcomes too, but few patients with BTC were included in those clinical trials: the ORR was 50% with selpercatinib and 66% with pralsetinib among 2 and 4 patients with BTC, respectively.^43,44^

Interestingly, drugs directed against HER2 demonstrated very different ORRs, ranging from 23% with pertuzumab and trastuzumab in the MyPathway trial to 41% with zanidatamab in patients with HER2-expressing BTC in the HERIZON–BTC-01 trial, regardless of HER2 score on immunohistochemistry (IHC).^30,31,34^ In the DESTINY–PanTumor02 trial, trastuzumab deruxtecan (T-DXd) yielded an ORR of 22% in the overall BTC cohort, but showed an ORR of 56.3% among patients with HER2 3+ BTC, irrespective of prior anti-HER2 therapy,^32^ likely suggesting that improving patients’ selection is the key to successful personalized targeted treatment. Indeed, the inclusion criteria for clinical trials with HER2-targeting agents vary, with more stringent participation criteria being associated with better outcomes. The MyPathway trial enrolled patients with *ERBB2* amplification based on NGS, fluorescence in situ hybridization (FISH), or chromogenic in situ hybridization, as well as those with HER2 overexpression based on IHC 3+ staining, with no specific requirements regarding the criteria used for scoring.^34^ By contrast, the HERIZON–BTC-01 included patients with *ERBB2*-amplified tumors confirmed by central in situ hybridization (ISH),^30^ while in the DESTINY–PanTumor02 basket study, only patients with HER2 3+/2+ on IHC were included, using the guidelines for scoring HER2 in gastric cancer.^32^ Notably, T-DXd yielded no objective responses among patients with HER2 2+ immunostaining only in the BTC cohort, suggesting that the bystander effect typical of antibody-drug conjugates may not be as effective in this type of tumor for reasons that still need to be elucidated.^32^ However, in the HERB trial, T-DXd showed preliminary signs of activity in a small cohort of 8 patients with HER2-low tumors (IHC 2+/FISH negative or IHC 1+), yielding one PR and 5 SD. However, responses were shorter lived in this subset of patients compared with those with HER2 3+/2+ FISH positive tumors (PFS: 3.5 mo vs. 5.1 mo); moreover, as the study was conducted on an all-Japanese population, these results cannot be easily transferred to Western patients.^52^ The fact that targeted agents are less effective for HER2 2+ BTCs has also been confirmed for zanidatamab, both in the HERIZON–BTC-01 trial (Figure 1A) and in the real-life setting.^30,31,53^ The 2025 update to the guidelines of the European Society of Medical Oncology (ESMO) for BTC recommends T-DXd or zanidatamab for patients with HER2 overexpression or amplification.^23^ However, it should be highlighted that the FDA has approved both T-DXd (in an agnostic fashion) and zanidatamab exclusively for patients with 3+ immunostaining. Likewise, the EMA has approved zanidatamab only in the case of HER2 3+ on IHC. Regarding the other anti-HER2 agents mentioned in Table 1, the association of pertuzumab and trastuzumab, which was previously recommended, has been removed from ESMO guidelines, but remains mentioned in the NCCN guidelines;^22–24^ the combination of FOLFOX and trastuzumab, albeit effective, was evaluated in an all-Korean population,^33^ therefore the results cannot be generalized to Western patients, and this regimen is not included either in the ESMO guidelines or in the NCCN guidelines.^22–24^

### Mechanisms of primary resistance

Despite the strong preclinical rationale, a subset of patients with actionable alterations are refractory to targeted agents. As far as *FGFR2* f/r are concerned, fusion partners seem to have an impact: in vitro, cells with *FGFR2–BICC1* fusions had different sensitivities to FGFR inhibitors, with those expressing fusion proteins without the kinase domain showing primary resistance;^54^ nonetheless, there is not enough evidence to support patients’ selection based on fusion partners. Moreover, co-mutations seem to play a crucial role in primary resistance to anti-FGFR drugs. A retrospective analysis of the screening cohort of the FIGHT-202 trial, which assessed pemigatinib in second line and beyond, highlighted that patients with mutations in tumor suppressor genes (eg, *TP53*, *CDKN2A/B*, and *PBMR1*) had a numerically lower ORR and a statistically significantly worse PFS.^55^ Similarly, in the FOENIX–CCA2 trial of futibatinib, patients with alterations in *CDKN2A/B* had a shorter PFS, albeit not significantly.^27^ Currently, there is not sufficient data to withhold FGFR-directed treatment in this subgroup of patients, but disease monitoring may need to be more stringent, as PD may be observed earlier. However, in the future, access to novel targeted agents could redefine drug sequencing, changing priorities in the decision algorithm. For example, protein arginine methyltransferase 5 (PRMT5) inhibitors, which have shown preliminary efficacy in tumors exhibiting *CDKN2A* loss and/or *methylthioadenosine phosphorylase* (*MTAP*) deletion as discussed later on, may move up in the treatment sequence.^56^

Concomitant molecular alterations may mediate primary resistance to anti-HER2 drugs as well: there is anecdotal evidence that tumors with co-occurring amplifications in other genes, such as *EGFR*, may respond exclusively to anti-EGFR therapies, suggesting that identification of the main oncogenic driver is essential, and definition of the priority of a driver in a case with co-occurring molecular alterations is mandatory.^57^ This is also strengthened by the data on the HER2 inhibitor neratinib, which has provided responses in cases with *ERBB2* mutations only in the absence of other mutations.^58^ Data from gastric cancer indicate that intra-tumor spatial heterogeneity in HER2 immunostaining is involved in resistance to HER2-directed drugs,^59^ but there is no evidence for this in BTC.

Data on the role of co-occurring molecular alterations in the primary resistance to other targeted therapies are lacking in BTC. Although growing evidence is present for other tumor types, this does not reflect in a change in indication for patients with BTC. For instance, in melanoma, loss of *PTEN*,^60^ cyclin-dependent kinase 4 mutations, cyclin D1 amplifications,^61^ and *NF1* mutations mediate resistance to BRAF inhibitors.^62^ In non-small cell lung cancer, there is anecdotal evidence that *KRAS* co-mutations might affect response to selpercatinib;^63^ moreover, *KEAP1*, *SMARCA4*, and *CDKN2A* mutations are associated with worse clinical outcomes during anti-*KRAS* G12C treatment.^64^ In patients with *NTRK* fusions receiving larotrectinib, concomitant *NTRK* G623R and G595R mutations interfere with drug binding, thus hindering its efficacy.^65^ Only in patients with *IDH1*-mutated BTC co-occurring alterations have failed to conclusively demonstrate an association with worse response to ivosidenib.^29,66^ As extended genomic profiling has only recently become a part of routine clinical practice, prospective data on the role of co-mutations in treatment resistance are lacking.

### PFS and OS

**FIGURE 2:**
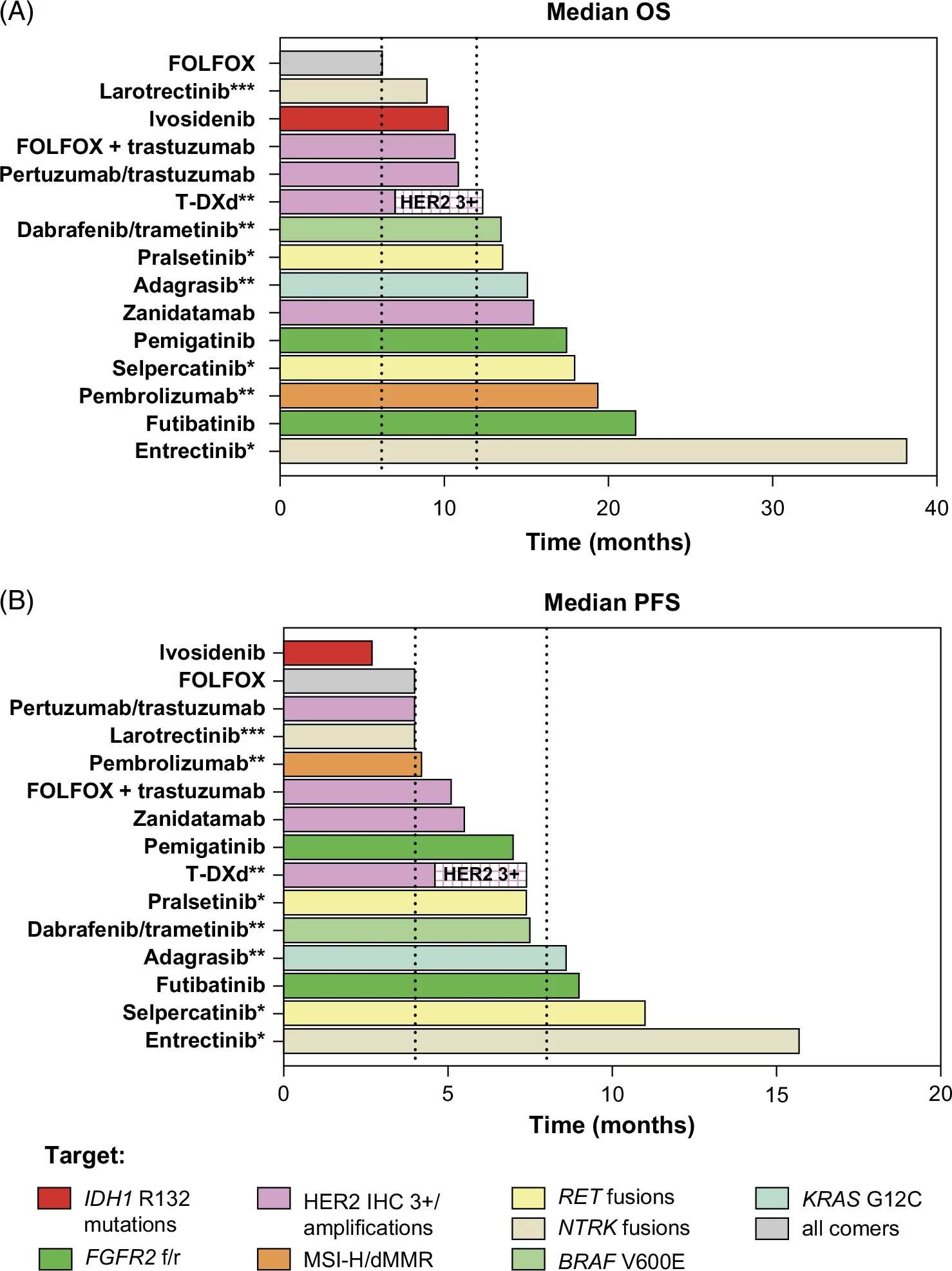
Clinical outcomes for patients with BTC receiving targeted treatment across key clinical trials in comparison to chemotherapy with FOLFOX.^17,25–45^ Panel A compares median OS values, while panel B compares median PFS values. Created with GraphPad and Adobe Illustrator. *Results relative to the overall cohort; **Results relative to the biliary cohort; ***Results relative to the gastrointestinal (non-colorectal) cohort. Abbreviations: dMMR, deficit in mismatch repair proteins; f/r, fusions/rearrangements; IHC, immunohistochemistry; MSI-H, microsatellite instability high; OS, overall survival; PFS, progression-free survival; T-DXd, trastuzumab deruxtecan.

All targeted agents provide a remarkable benefit in OS, with at least a 3-month advantage in median OS (from 9 mo to over 3 y) compared with the one observed with FOLFOX (6 mo) (Figure 2A). Moreover, most targeted drugs yielded an improvement in PFS, surpassing the 4-month benchmark set by FOLFOX, with the exception of ivosidenib, which yielded a median PFS of 2.7 months (Figure 2B). While the PFS rate at 6 months was 32% with both treatment regimens, the PFS rates at 12 months were 22% and 8.6% for ivosidenib and FOLFOX, respectively, suggesting that there is a subset of patients that derives prolonged benefit from this targeted treatment.^17,28,29^ Besides, in a retrospective observational trial, outcomes of patients receiving ivosidenib were compared with a historical cohort of patients with *IDH1*-mutated CCA treated with FOLFOX. Median PFS was 6.9 months versus 2.1 months, respectively, reinforcing the notion that ivosidenib is a valid option in this setting.^67^

Interestingly, pembrolizumab, the association of pertuzumab and trastuzumab, and larotrectinib demonstrated a similar PFS to FOLFOX, but a longer OS benefit, yielding a median OS of 19.4, 10.9 months, and 9 months, respectively; long-term outcomes were improved as well, with a 36-month OS rate of 30.3% for single-agent immunotherapy, a 24-month OS rate of 14% for larotrectinib, and a 12-month OS rate of 50% for the anti-HER2 combination.^34–37,40^ This could be due to the fact that patients on targeted therapy receive more subsequent lines of treatment, as supported by real-world data as well.^46^

Indeed, targeted treatments seem to have a positive impact on QoL, and it is reasonable to believe that patients may still be fit for further treatment after PD. In the ABC-06 trial, at a 4-month follow-up, FOLFOX did not determine a worsening of the parameters assessed by the European Organization for Research and Treatment of Cancer (EORTC) Quality-of-life Questionnaire-Core 30 (QLQ-C30), including global and physical health, role, social function and emotional scale, pain and nausea; by contrast, patients in the control arm experienced a statistically significant worsening of all of these parameters.^68^ These results support the use of further lines of treatment after PD as a first line in patients who are fit. For those with a druggable alteration, targeted drugs represent an opportunity as they not only managed to maintain QoL for long periods of time, but also determined an improvement in certain domains. In the ClarIDHy trial of ivosidenib, QoL was maintained during treatment, even among patients who received this therapy for over a year; according to the EORTC QLQ-C30 assessment, ivosidenib preserved physical functioning, with a least-squares mean change from baseline of −0.2 versus −12.6 with placebo after 2 months; after 1 month of treatment, targeted therapy was also favored in the QLQ-C30 pain scale, emotional and cognitive functioning, dyspnea, QLQ-BIL21 anxiety and tiredness.^29,69^ Pembrolizumab was also well tolerated in the long term, with QLQ-C30 global health scale in the overall cohort improving by a mean of 3 points during the first 9 weeks and then remaining stable or improving through 2 years of treatment.^70^ Similarly, in the FOENIX–CCA2 trial, futibatinib led to sustained QoL results for up to 9 months from treatment start, and performance status was maintained or improved compared with baseline; only QLQ-C30 constipation worsened beyond the set threshold of −10 points after 4 cycles of treatment.^27,71^ Moreover, QoL outcomes correlated with treatment response. Patients who achieved disease control with pemigatinib had maintained QLQ-C30 overall health status and emotional functioning during the first 4 months of treatment, as well as decreased QLQ-BIL21 pain and anxiety, while those with PD saw a worsening in all of these domains.^72^ Likewise, in the HERIZON–BTC-01 trial, patients who responded to zanidatamab reported an improvement in QoL according to the EQ-5D-5L scale greater than those with SD and PD; in particular, >90% of patients with PR reported no problem in conducting their usual activities at the best response, compared with 68% and 53% of those with SD and PD, respectively. In comparison to baseline, there was an improvement in pain by 10 and 5 points on the visual analogue scale among patients who had CR/PR and those who had SD, respectively, while in those who had PD, pain worsened by 10 points.^73^ Patients treated with pembrolizumab also had better QoL outcomes when responding to treatment, with patients having a CR or a PR representing the largest proportion of improved QLQ-C30 outcomes after 3 cycles of immunotherapy.^70^ This reinforces the importance of identifying drugs that can induce tumor shrinkage even in the palliative setting. However, it is noteworthy that all patients treated with pemigatinib had a decline in QLQ-C30 social and role functioning and an increase in QLQ-BIL21 treatment side effects,^72^ underscoring the necessity of continued shared decision-making during treatment, in relation to each patient’s priorities. Data on QoL for other targeted drugs in patients with BTC are lacking and derive from their use in other populations, but positive findings are consistent across all of them, when available. Larotrectinib determined an improvement in health-related QoL in 75% of adult patients after 2 months of treatment, with a median duration of sustained improvement of 1 year in QLQ-C30 global health scale.^74^ The median time to physical function improvement with selpercatinib was 4 months, and it was sustained for 12 months.^75^ Entrectinib also guaranteed a sustained QLQ-C30 global health status and a clinically meaningful improvement (≥10 points) in role functioning, fatigue, and appetite loss, with diarrhea being the only worsening symptom after 13 cycles of treatment.^76^

In light of these results, it is sensible to conclude that part of the clinical benefit derived from targeted therapy lies in a combination of factors, including disease stabilization, reduced toxicity, and increased life expectancy, with the possibility of having access to additional lines of treatment. This is reflected in an improvement in OS observed across all studies on targeted agents, with T-DXd, dabrafenib/trametinib, zanidatamab, adagrasib, and pemigatinib yielding a median OS even longer than a year, futibatinib of almost 2 years, and entrectinib of approximately 3 years (Figure 2A).

**FIGURE 3:**
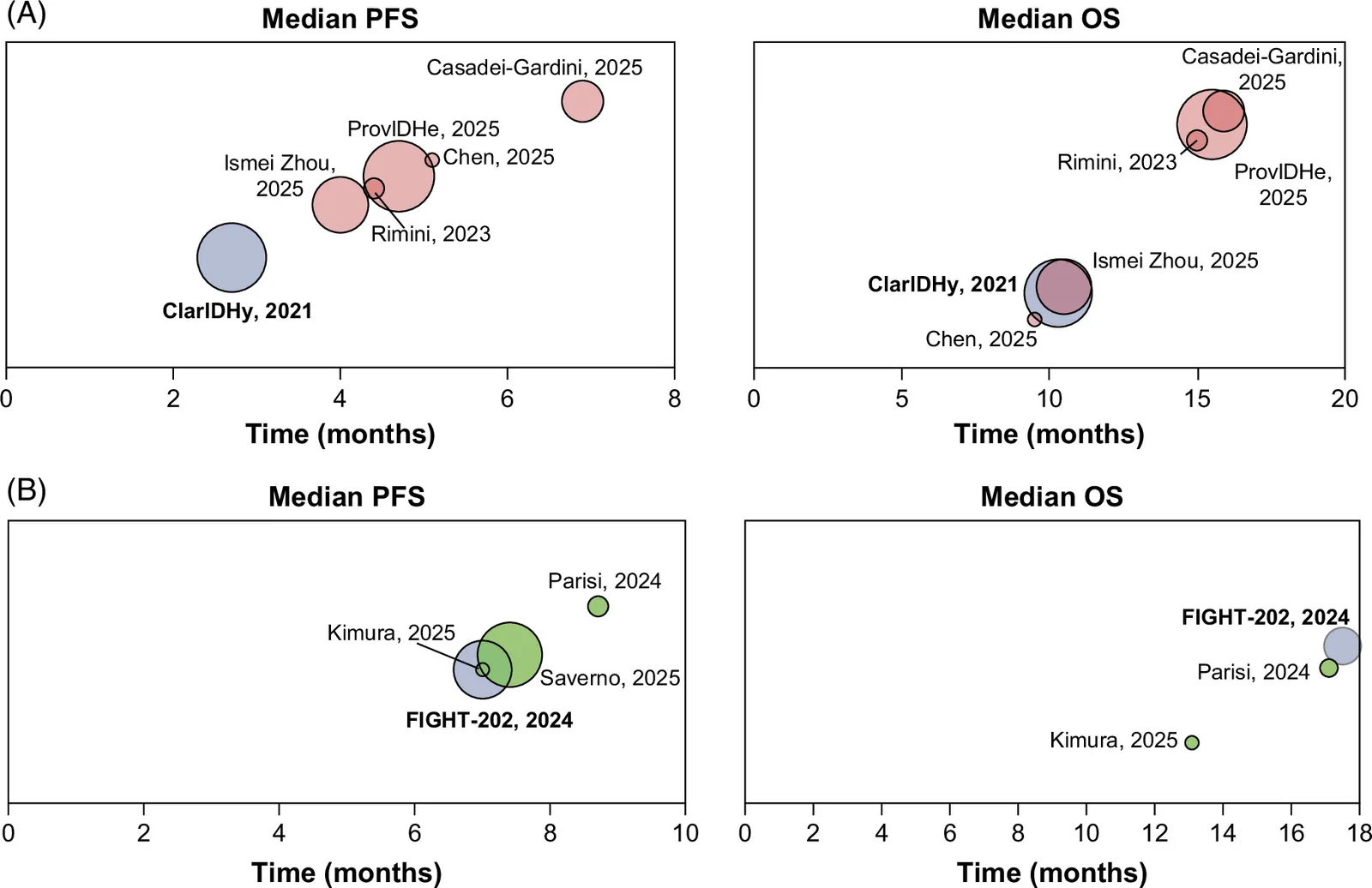
Clinical outcomes of ivosidenib and pemigatinib in real-life studies compared with clinical trials.^26,28,29,67,77–84^ Panel (A) focuses on ivosidenib, while panel (B) focuses on pemigatinib. Reference clinical trials (ClarIDHy and FIGHT-202) are in blue. Bubble size indicates the dimension of the study population. Created with GraphPad and Adobe Illustrator. Abbreviations: OS, overall survival; PFS, progression-free survival.

Real-life studies are needed to establish the benefit of targeted treatments across different populations. Currently, real-world data is available for ivosidenib and pemigatinib, as they are the ones that were approved first and have a more widespread availability. In general, in the real-life setting, both of them have demonstrated similar or better results than those observed in the ClarIDHy and FIGHT-202 trials, both in Western and Asian patients; the only exception is represented by the Japanese population, where pemigatinib has shown a worse median OS than anticipated (13 mo vs. 17.5 mo) (Figure 3). Preliminary evidence is also emerging for anti-HER2 agents. In particular, in a retrospective cohort of 20 patients, zanidatamab demonstrated a median PFS of 6.7 months and a 24-month OS rate of 73%;^53^ in a single-center retrospective study of 37 patients, including 5 with BTC, T-DXd showed a median PFS of 7.3 months and a median OS of 10.2 months.^85^

### Safety

The safety profile of targeted therapies is different from that observed with chemotherapy, and needs to be factored into the assessment of patients’ eligibility for targeted agents. This is not meant to be a comprehensive list of possible TRAEs, but a highlight of the ones that may influence treatment choice. While skin and nail toxicities, as well as alopecia, commonly observed with anti-FGFR drugs, can affect QoL,^25–27^ they will rarely represent an absolute contraindication to treatment. By contrast, the risk of potential cardiac, respiratory, and neurological or ocular toxicity needs to be carefully weighed in.

### Cardiac toxicity

Anti-HER2 agents may determine a reduction in left ventricular ejection fraction (LVEF). This AE was observed in 6% of the patients treated with zanidatamab, while it was not reported in the DESTINY–PanTumor02 trial, although data deriving from breast cancer trials suggest an equally low incidence (1%).^30,32,73^ In HERIZON–BTC-01, all cases were asymptomatic, and patients showed an improvement or a complete resolution of the event.^30^ Nonetheless, a baseline echocardiogram and subsequent monitoring are mandatory. Anti-HER2 drugs were never evaluated in patients with LVEF <50%, therefore those with pre-existing cardiac comorbidities may not be suitable candidates for this type of treatment. Similarly, in the ROAR trial, trametinib, in combination with dabrafenib, was associated with a risk of cardiac-related events (6.7%), thus echocardiographic monitoring is recommended during treatment; notably, patients with significant cardiac history (eg, chronic heart failure of classes II–IV according to New York Heart Association, recent acute coronary syndrome, uncontrolled clinically significant arrhythmias, uncontrolled hypertension) were excluded from the study.^38,39^

QTc interval prolongation is an AE observed with adagrasib (12.7%),^45^ selpercatinib (11%),^43^ ivosidenib (6%–10%),^28,29,86^ and entrectinib (1%), with the latter being associated with a risk of cardiac failure as well (2%–3%), regardless of prior history of cardiac disease.^41^ Ahead of commencing any of these drugs, patients should undergo electrocardiogram evaluation, with values >500 ms representing an absolute contraindication to treatment; monitoring of this parameter during treatment is also crucial, as grade 2–3 QTc prolongation (>480 ms) warrants the interruption of treatment due to the risk of developing serious arrhythmias.

### Pulmonary toxicity

Pulmonary AEs have been observed with anti-HER2 drugs. In the DESTINY–PanTumor02 trial, interstitial lung disease (ILD) occurred in 10.5% of patients, including 3 fatal cases.^32^ A pooled analysis of 9 studies across multiple cancer types showed an overall incidence of 15.4% with T-DXd, with 77% of the patients having a grades 1–2 AEs and >85% experiencing it during the first year of treatment; among other factors, presence of lung comorbidities, moderate to severe renal impairment, and baseline oxygen saturation <95% determined an increased risk of developing ILD.^87^ A pooled analysis of trials on T-DXd showed that re-treatment after grade 1 ILD is feasible, with one-third of patients experiencing a recurrence, but always low-grade, stressing the importance of monitoring patients for the development of this AE to increase early detection rates.^88^ Only one case of pneumonitis was registered with zanidatamab, but it led to treatment discontinuation.^30^ Other drugs associated with ILD include pembrolizumab (2.6%) and the combination of dabrafenib and trametinib (2.4%).^38,39^

### Neurological and ocular toxicity

NTRK inhibitors are associated with neurological and cognitive side effects. Dizziness is the most common one, with either larotrectinib or entrectinib.^40,41^ In a real-world study including 96 patients, it was identified in 41% of the cases, being concomitant to ataxia in 6 patients; other common side effects included weight gain secondary to hyperphagia (51%) regardless of weight at baseline and withdrawal pain (34%).^89^ Other neurocognitive AEs have an incidence <5% and include alterations in mental status or mood and agitation.^40,41^

Ocular toxicity is typical of FGFR inhibitors. It encompasses a range of possible AEs such as xerophthalmia, central serous retinopathy, and retinal detachment. Retinal damage has been observed in 4% and 8% of the patients in the FIGHT-202 and in the FOENIX–CCA2 trial, respectively.^25–27^ Due to the potential severity of these AEs, an ophthalmological evaluation at baseline and then every 6–12 weeks is mandatory, meaning that patients with pre-existing ocular comorbidities may not be suitable for treatment with this class of drugs.

## GENOMIC TESTING

Current guidelines recommend that an NGS analysis be performed at diagnosis of advanced BTC for all patients who are fit for pSACT. Both DNA and RNA assays should be performed to capture the full array of alterations of interest, with single-nucleotide variants and amplifications being interrogated on the DNA level, while f/r on the RNA level.^22–24^

The importance of extended genomic profiling lies in the possibility of detecting the full spectrum of co-occurring molecular alterations. In fact, not only are targetable alterations not mutually exclusive, but concomitant mutations also play a role in resistance to treatment. However, access to NGS is burdened by several challenges, related to the availability of adequate laboratory technologies and elevated costs, leading to a lack of reimbursement by local national healthcare systems in several countries.^21^

**FIGURE 4:**
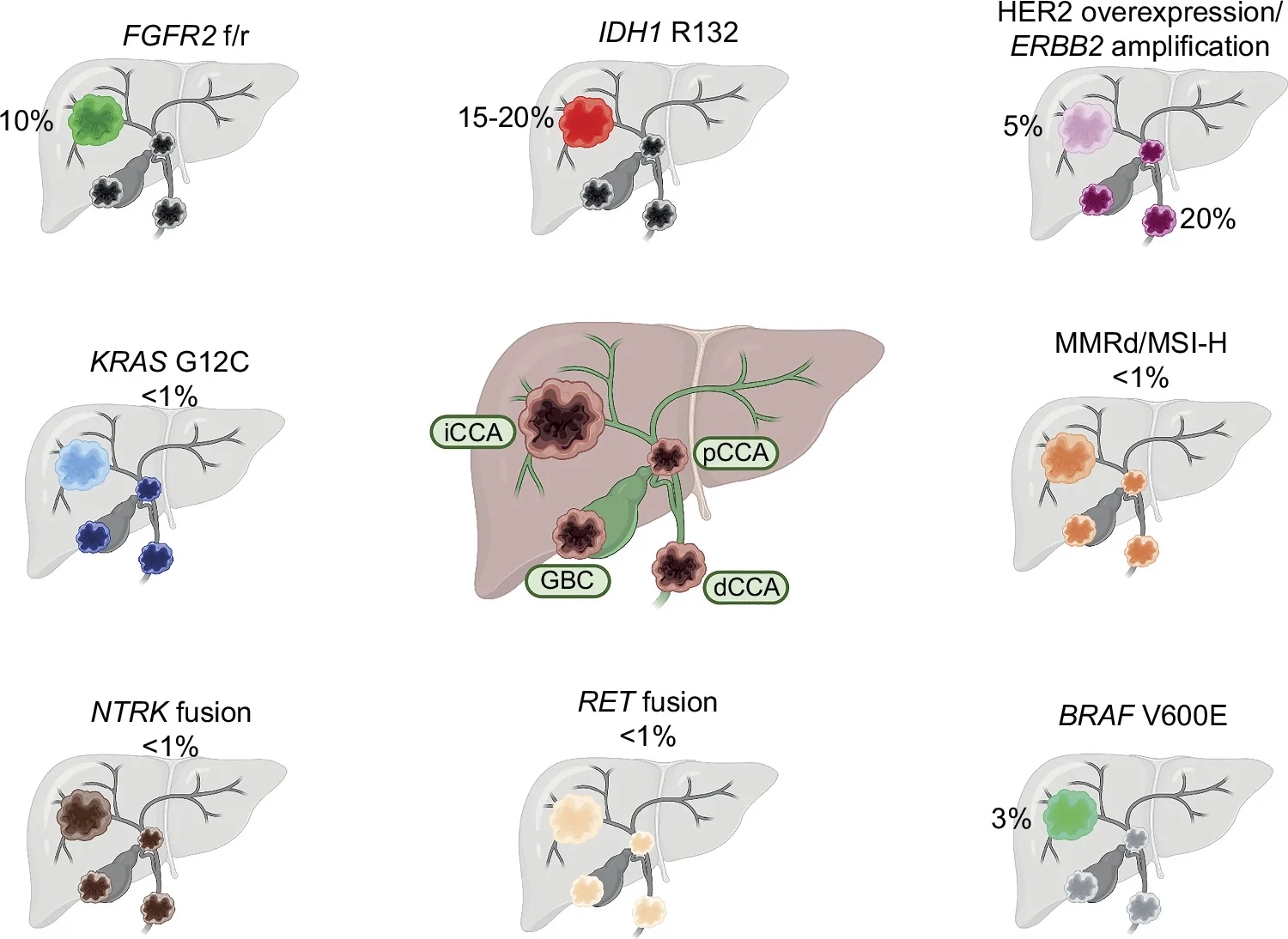
Frequency of actionable molecular alterations in relation to BTC subtypes.^21,90^ For each alteration, the anatomical subtypes in which it can be found are colored. If the prevalence is higher for a specific subtype compared with others, they are represented on a gradient, with the darker color indicating a higher frequency. Created in BioRender. Abbreviations: dCCA, distal cholangiocarcinoma; f/r, fusion or rearrangement; GBC, gallbladder cancer; iCCA, intrahepatic cholangiocarcinoma; MMRd, deficit in mismatch repair proteins; MSI-H, microsatellite instability high; pCCA, perihilar cholangiocarcinoma.

When NGS is not available, a selected number of genes should be investigated through single-gene analysis to guarantee access to matched targeted drugs. The gene panel should include at least *IDH1*, *FGFR2*, *ERRB2*, *KRAS*, *BRAF*, *RET*, *NTRK*, and mismatch repair (MMR) genes.^22,23^ Of note, molecular alterations are not equally distributed across BTC subtypes. Therefore, in case of lack of resources, specific ones should be prioritized according to prevalence (Figure 4).

In particular, patients with intrahepatic CCA should always be tested for *IDH1* mutations and *FGFR2* f/r, which can be evaluated with polymerase chain reaction (PCR) and FISH, respectively, in the absence of access to NGS technologies. Nonetheless, RNA sequencing remains preferable for FGFR2 f/r: even though results are usually comparable to FISH,^91,92^ the latter is not informative for intrachromosomal rearrangements, which represent half of all *FGFR2* rearrangements in BTC; it also lacks the ability to detect complex genomic alterations, transcribed and in-frame fusions, therefore being a more limited testing technique compared with RNA sequencing.^93,94^ In contrast, DNA-based NGS has a lower sensitivity, with false-negative rates of ~30% according to recently published data.^95^ However, it has the ability to detect *FGFR2* mutations, which may represent mechanisms of resistance to certain FGFR inhibitors.

On the other hand, in patients with extrahepatic CCA or GBC, HER2 status should always be determined.^22,23^ There is a knowledge gap with regard to the optimal scoring method for HER2 on IHC in BTC, as well as the concordance between results on immunostaining and other techniques. So far, the IHC scoring system used for gastric cancer seems to be the one most fitting for BTC.^23,96^ According to this method, different from breast cancer, complete membranous staining for HER2 is not necessary, and only the basolateral membrane is considered for scoring, as the luminal surface may not be informative and may fail to stain in gastric cancers.^97^ However, its results do not necessarily correlate with those obtained with ISH or DNA sequencing. A translational analysis of the HERIZON–BTC-01 trial showed a 94% concordance rate between HER2 IHC 3+ and amplification on ISH.^98^ However, there is less evidence on the concordance between copy number variant (CNV) scores from DNA sequencing, either on blood or tissue, and ISH/IHC techniques. In the same analysis, the concordance between gene amplification on ISH and DNA sequencing on liquid biopsy using an FDA-approved panel (Guardant360) was 59%.^98^ The Guardant360 reports CNV between 2.5 and 4.0 as ++ and CNV >4.0 as +++ amplifications, corresponding to the 50th–90th and >90th percentile, respectively, of all CNV in the Guardant360 database.^99^ This provides a semi-quantitative assessment of the level of amplification of the gene, without identifying a clear cut-off. To our knowledge, the only study that has reported this information thus far is the KCSG–HB19-14 trial, where a gene copy number ≥6 was used to define *ERBB2* amplification.^33^ Interestingly, despite all 34 participants having HER2-positive status confirmed by IHC (3+ or 2+ confirmed by FISH), only 31% had an amplification detected on NGS; moreover, only one patient with HER2 2+ status confirmed on FISH showed an amplification on NGS.^33^ Therefore, even though DNA-based multi-gene panels are becoming more widespread in clinical practice, they should be used with caution to determine eligibility to anti-HER2 agents, given the lack of evidence of a direct correspondence between NGS and IHC results, and the latter should be preferred, as it is the criterion referenced for approvals by regulatory bodies.

Other molecular alterations are rarer in BTC, but are worthy of assessment. If they are not included in a multi-gene panel, consideration should be given to single-gene testing, even though this is usually not recommended, as it delays results and reduces tissue optimization.^100^ PCR can be informative of *BRAF* and *KRAS* mutations. *NTRK* fusions should be investigated by RNA-sequencing: ESMO guidelines recommend that NGS panels are the most cost-effective approach, considering the low prevalence of *NTRK* fusions and the need to investigate other alterations at the same time.^101^ For instance, RNA-sequencing can also provide information on *FGFR2* and *RET* fusions. However, IHC is quick and cheap, requires little material, and has high sensitivity and specificity for *NTRK* fusions.^101^ If NGS testing is not easily accessible, a 2-step approach where only IHC-positive samples are subjected to RNA-sequencing has been proposed by multiple guidelines in tumors with an expected prevalence of *NTRK* fusions <1%,^101,102^ and seems feasible in clinical practice for pancreatobiliary malignancies.^51^

Lastly, microsatellite instability (MSI) and/or MMR should still be investigated to determine eligibility for pembrolizumab, although the clinical relevance is now reduced in BTC, given that all patients receive immunotherapy in first line as part of the standard of care.^22–24^ However, MMR deficiency (dMMR)/MSI-high (MSI-H) features have repercussions on patients’ management, usually prompting genetic testing for Lynch syndrome, with implications for high-risk screening. MMR genes can be studied both through NGS analysis to determine MSI-high status or through IHC to determine MMR deficiency. Concordance between the 2 analyses in colorectal cancer is close to 100%, but discordance may be observed in other tumor types.^103–105^ In BTC, there is no evidence regarding concordance between IHC and NGS. In the KEYNOTE-158 trial, both patients with MSI-H and dMMR tumors were included, therefore either technique can be utilized.^36^

Even when NGS analysis is available, challenges may arise due to a lack of tissue, as acquisition can be difficult due to anatomical location, especially for perihilar or distal CCA. Liquid biopsy is a non-invasive tool that may help bypass these issues, while also being more informative of intra-tumor heterogeneity than tissue biopsy. Although it has not been integrated into clinical practice yet, evidence points to its feasibility, with possibly different yields based on BTC anatomical subtype. In a cohort of 102 patients, circulating tumor DNA (ctDNA) analysis demonstrated a sensitivity rate of 84% and a positive predictive value of 79%. However, at subgroup analysis, sensitivity was >90% for intrahepatic CCAs and GBC, while it dropped to 56% for perihilar and distal CCAs; positive predictive value was consistent across all anatomical subtypes of BTC. These results confirm the reliability of liquid biopsy as a diagnostic tool, but suggest that extrahepatic CCAs may be associated with lower shedding of circulating tumor cells.^106^ Yield differs according to specific molecular alterations, as well. In a large cohort of over 1600 patients, ctDNA analysis demonstrated a concordance rate with tissue analysis of >85% for *IDH1* and *BRAF* mutations, but of only 18% for *FGFR2* fusions.^107^ Newer panels, such as the TruSight Illumina panel utilized in the FOENIX–CCA2 study, have improved these outcomes, increasing concordance rates up to 85%,^27^ but still need external validation.

## FUTURE PERSPECTIVES

### Overcoming secondary resistance mechanisms

Despite the efficacy of targeted agents, patients inevitably progress, with most drugs guaranteeing a median duration of response (DOR) of ~10 months among those who achieve an objective response (Figure 1C). Mechanisms of secondary resistance differ across targets and may be due to either on-target alterations, which may be responsive to novel drugs, or to off-target mechanisms that may be overcome by new therapeutic combinations.

Resistance to FGFR inhibitors is associated with de novo secondary *FGFR2* mutations, most frequently V565 and N550.^108^ Tinengotinib is a reversible multikinase inhibitor capable of bypassing on-target mutations, as its binding site is located outside of the active site. In a phase II single-arm trial, it yielded a DCR of 94.7% among patients resistant to FGFR inhibitors, with a median PFS of 5 months and a 1-year OS rate of 65%. It was well tolerated, with hypertension (25%), palmar-plantar erythrodysaesthesia syndrome, diarrhea, and stomatitis (6.3% each) being the most common grade 3 AEs.^109,110^ A phase III randomized trial investigating its efficacy in comparison to chemotherapy after PD to at least 2 previous lines of treatment, including only one FDA-approved FGFR inhibitor, is ongoing.^111^ By contrast, lirafugratinib is a selective FGFR2 inhibitor capable of bypassing acquired resistance mechanisms, including N549 and V564 mutations. ORR and DCR were 15% and 80%, respectively, among patients who received previous anti-FGFR2 therapy, with a median DOR of 5 months.^112^ Activations of other molecular pathways as a consequence of off-target mutations (eg, *RAS* mutations activating the EGFR signaling pathway) have led to evaluating treatment combinations, such as pemigatinib and the EGFR inhibitor afatinib (NCT06302621). Lastly, FGFR inhibitors may lead to modifications in the tumor microenvironment, suggesting that there may be scope to investigate their use in association with immune checkpoint inhibitors.^113^ In a Chinese phase I/II trial, in an unselected population, tinengotinib and atezolizumab yielded an ORR of 33%; a phase II study assessing the combination of atezolizumab and the FGFR inhibitor deranzantinib in patients with FGFR2 f/r is ongoing (NCT05174650).

Secondary resistance to ivosidenib in *IDH1*-mutated BTCs is poorly understood, with evidence deriving from small case series. It seems to be mediated by oncogenic *IDH2* mutations, isoform switching, and new-onset *IDH1* mutations that impair drug binding.^114,115^ For instance, a second site mutation inducing the IDH1 R132C/S280F form is capable of both blocking ivosidenib binding and inducing a higher rate of 2-HG production.^116^ Analysis of pre- and on-treatment tissue biopsies also showed an activation of PI3K/AKT/mTOR signaling in rapid progressors without any genetic alterations that might justify this, possibly due to 2-HG activating this pathway.^50^ No drugs capable of overcoming these resistance mechanisms are available in clinical practice.

Data on resistance to anti-HER2 therapies derive mostly from other gastrointestinal tumor types; it involves activation of signaling pathways capable of bypassing HER2, such as PI3K/AKT/mTOR, progressive loss of HER2 expression, de novo HER2/3 mutations, and masking of the HER2 epitope due to excessive mucin and hyaluronic acid production.^117^ In addition, treatment resistance may be due to concomitant molecular alterations. In particular, *CCNE1* amplification leads to worse PFS to trastuzumab in gastric cancer.^118^ In vitro evidence indicates that cyclin E overexpression, secondary to *CCNE1* amplification, induces resistance to T-DXd; in vivo, sensitivity to T-DXd was rescued by adavosertib, a Wee1 kinase inhibitor, prolonging event-free survival in patient-derived xenografts of gastrointestinal cancers.^119^ With specific regard to BTC, in vitro studies indicate that sensitivity to trastuzumab can be restored by bosutinib, an inhibitor of Src, which is a proto-oncogene that upregulates AKT and may link the crosstalk between MET and EGFR.^120^ However, there are no clinical data to support its efficacy in clinical practice.

There is a lack of data on acquired resistance to other targeted treatments in BTC, with evidence derived from other tumor types.

In a cohort of 38 patients, resistance to adagrasib was found to be due to novel *KRAS* mutations within the binding site of the drug, including Y96C, H95D/Q/R, and R68S, as well as other new-onset *KRAS* mutations, such as G12D/V/R, Q61H, and G13D. Off-target mechanisms included activating *NRAS* mutations.^121^ Compounds targeting all RAS isoforms are under investigation in patients who did not receive previous KRAS inhibitors, as discussed later on. In pretreated patients, their efficacy might be hindered by the presence of other concomitant resistance mechanisms; in particular, in this study, 41% of patients developed more than one mechanism of resistance to adagrasib. Other bypass mechanisms included *MET* amplifications; *NF1*, *PTEN*, *NRAS*, *BRAF*, *MAP2K1*, and *RET* mutations; *ALK*, *RET*, *BRAF*, *RAF1*, and *FGFR3* fusions; besides, adeno-to-squamous transition was observed in patients with non-small cell lung cancer, in the absence of other alterations identified.^121^

In a case series comprising 18 patients, NGS analysis conducted on post-progression biopsies from patients treated with larotrectinib or entrectinib revealed *NTRK* mutations on the solvent front (G623E/R, G595R) as the most common on-target resistance mechanism.^122^ Repotrectinib is a tyrosine kinase inhibitor (TKI) directed against TRK and ROS1 with a structure capable of overcoming these alterations. In the phase I/II TRIDENT-1 basket trial, ORR was similar among TKI-pretreated and naïve patients (50% and 58%, respectively); notably, TKI-pretreated patients with solvent front mutations had an ORR of 60%. Among 3 patients with CCA, 1 was TKI-naïve and achieved SD, while 2 were pretreated and an SD and a PR were observed.^123^

Lastly, resistance to RET inhibitors is polyclonal and is mostly related to the activation of the MAPK pathway, through *RAS* or *BRAF* mutations, and *MET* or *FGFR1* amplifications.^63^

In the future, the increasing evidence regarding resistance mechanisms to treatment and the availability of new targeted agents may pave the way for the standardization of longitudinal molecular analysis during pSACT aimed at tailoring treatment even further.

### Targeted therapies beyond the second-line setting

Considering the upsides associated with targeted therapy, the following step is determining whether they could be relocated in the treatment algorithm to an earlier setting, with the goal of improving clinical outcomes and guaranteeing access to a higher proportion of patients. There is no solid data in BTC on how many patients miss the opportunity to access targeted treatment due to rapid PD. In the MOSCATO trial, which encompassed multiple tumor types, 15% of patients with an actionable target did not receive matched treatment due to clinical deterioration.^124^ In addition, given the higher ORR achieved with targeted therapies compared with chemoimmunotherapy, accessing these strategies in first-line may increase downstaging rates for locally advanced cancers, with improved chances to proceed to curative surgery.

If we consider the more common targetable molecular alterations in BTC, *ERBB2* amplifications were found to be associated with more aggressive features (larger tumor size, nodal metastases, and advanced stage).^125^ In the translational analysis of the TOPAZ-1 trial, *ERBB2* amplifications were identified in 9% of non-long-term survivors versus 4% of long-term survivors, and in an exploratory analysis of the same study, these patients did not have a PFS or OS benefit from the addition of immunotherapy to chemotherapy.^126,127^ Recent evidence points to HER2-positivity being a negative prognostic factor, with patients showing both a shorter PFS to first-line pSACT and worse OS outcomes, which could be rescued by targeted therapies.^128^ This is the rationale behind clinical trials assessing anti-HER2 agents in first-line. In a phase II trial, first-line zanidatamab in combination with cisplatin and gemcitabine yielded an ORR of 43% and a DCR of 86% with a median PFS of 9.9 months; OS data are not mature yet.^129^ However, these results support the randomized phase III HERIZON–BTC-302 trial of zanidatamab in combination with cisplatin and gemcitabine ± immunotherapy, which is underway.^130^ Furthermore, the DESTINY-Biliary Tract Cancer-01 is evaluating T-DXd alone or in association with the bispecific antibody rilvegostomig (directed against TIGIT and PD-1) compared with standard-of-care cisplatin, gemcitabine, and durvalumab.^131^ Both clinical trials will also include patients with HER2 2+ BTC, with FISH confirmation required in the former to define HER2-positivity, but not in the latter.

Similarly, the safety of ivosidenib in combination with first-line cisplatin, gemcitabine, and durvalumab is currently being assessed in a phase Ib/II trial.^132^ This treatment combination has a preclinical rationale, as IDH1 inhibition is known to restore sensitivity to antitumor immune response.^133^ Besides, it needs to be stressed that ivosidenib cannot be offered to patients with rapid PD in the attempt to improve symptom control and reduce disease burden, because it is a cytostatic drug.^50^ Although *IDH1* mutations do not have a clear prognostic value,^134^ it is possible that patients with *IDH1*-mutated BTC may miss the opportunity to receive matched targeted treatment due to clinical deterioration. For this reason, there is scope to investigate the role of ivosidenib in the first-line setting, either as an upfront treatment or as maintenance therapy after a course of chemoimmunotherapy, especially if a deep response to first-line pSACT is observed. In this regard, the ongoing phase III SAFIR-ABC10 umbrella trial is set out to determine the efficacy of introducing targeted therapy in first line, after 4 cycles of chemoimmunotherapy, versus continuing first-line pSACT until PD or unacceptable toxicity as per current standard of care.^135^

On the other hand, *FGFR2* f/r seem to be favorable prognostic factors, showing better OS outcomes regardless of access to targeted therapy.^136,137^ Phase III trials of FGFR inhibitors in first line include the FIGHT-302 of pemigatinib, the FOENIX-CCA3 of futibatinib (NCT04093362), and the PROOF 301 of infigratinib, but they have all been discontinued prematurely due to slow accrual.^138,139^ However, considering the apparently more indolent nature of BTCs harboring *FGFR2* f/r, a sequencing strategy aimed at increasing therapeutic choices for these patients might be considered. There is no data on whether a treatment sequence or an upfront strategy is more effective.

As far as resectable disease is concerned, there are no ongoing clinical trials for targeted therapies in this setting. Neoadjuvant treatment is not a part of the therapeutic algorithm for BTC. However, it may be worthwhile exploring the role of preoperative targeted agents, as preliminary evidence suggests that neoadjuvant chemotherapy increases R0 rates and may have a positive impact on OS outcomes.^140^ Drugs that have demonstrated promising cytoreductive activity, with ORRs ≥50%, include zanidatamab and T-DXd for patients with HER2 3+ BTC, entrectinib and pralsetinib (Figure 1A).

### Newly identified actionable targets

The array of potentially actionable alterations for patients with BTC is growing at a fast pace, with numerous ongoing clinical trials exploring the safety and efficacy of new targeted drugs (Table 3).

**TABLE 3:** Newly identified actionable targets in patients with biliary tract cancer

Trials with available data for patients with BTC								
Actionable target	Frequency	Drug	Phase of trial	Patients with BTC (n)	ORR (%)	DCR (%)	Median PFS (mo)	Median OS (mo)
*ERBB2* mutations	1%–2%	Neratinib^58^**	II	25	16	24	2.8	5.4
		Zongertinib^141^***	Ia	1	17.2	72.4	NR	NR
HRD	Up to 20%	Olaparib^142^**	II	31	6.5	74.2	16.7 wk	11.2
*MDM2* amplifications	5%–10%	Brigimadlin ± ezabenlimab^142^**	Ia/Ib	16	43.8	87.5	NR	NR
*MTAP* deletion	15%	AMG 193^56^*	I	4	21.4	54.8	NR	NR
**Other ongoing clinical trials**								
**Actionable target**	**Frequency**	**Drug**	**Phase of trial**	**NCT number**		**Status**	**Primary endpoint**	
*KRAS* G12D	40% of all *KRAS* mutations	Zoldonrasib	I/Ib	NCT06040541		Recruiting	Safety, DLT	
*RAS* mutations	KRAS: 10%–30% NRAS, HRAS: 3%	Daraxonrasib	I/Ib	NCT05379985		Recruiting	Safety, DLT	
*MDM2* amplifications	5%–10%	Brigimadlin^144^	IIa/IIb	NCT05512377		Active, not recruiting	ORR per BICR	
Claudin 18.2	5% (GBC: 15%)	Sonesitatug vedotin^145^	II	NCT06219941		Recruiting	Safety, ORR	
Claudin 18.2		Spevatamig	I/II	NCT05482893		Recruting	DLT, MTD, safety	
*TP53* Y220C	2% of all *TP53* mutations	Rezatapopt^146^	I/II	NCT04585750		Recruiting	ORR per BICR in the ovarian cancer cohort and across cohorts	

*Overall cohort; **Biliary cohort; ***Gastrointestinal cohort

Abbreviations: BICR, blind independent central review; BTC, biliary tract cancer; DCR, disease control rate; DLT, dose-limiting toxicity; GBC, gallbladder cancer; HRD, homologous repair deficiency; MTD, maximum tolerated dose; NR, not reported; ORR, objective response rate; OS, overall survival; PFS, progression-free survival.

### ERBB2 mutations

*ERBB2* mutations are rarer than amplifications, occurring in 1%–2% of the patients.^147^ Neratinib is an irreversible pan-HER oral TKI. In the phase 2 SUMMIT basket trial, the BTC cohort included 25 patients. The ORR was 16% and the DCR was 24%, with a median PFS of 2.8 months, and better outcomes were observed among patients with GBC. Interestingly, responses were associated with a lack of co-occurring mutations, confirming that the selection of the main driver is the key factor for the success of targeted therapies. Serious TRAEs were observed in 8% of the cases.^58^ Zongertinib is another irreversible TKI, but due to its selective affinity for HER2, it should have a more manageable safety profile. In a phase Ia trial, in a cohort of 33 patients with HER2-altered gastrointestinal cancers, including 4 with BTC, ORR was 21% with a DCR of 82% and a median PFS of 124 days.^141^ Targeted therapies directed against *ERBB2* mutations are currently not approved by any regulatory body nor recommended by international guidelines.

### Homologous repair deficiency

The most frequently mutated genes involved in DNA repair in BTC include *ARID1A* (10%–20%),^147,148^ *ATM* (5%–8%),^147^ and *BRCA1/2* (3%).^90,149^ Although patients with homologous repair deficiency (HRD) (germline or somatic) were documented to have longer PFS and OS when receiving platinum-based chemotherapy,^149^ more recent evidence has shown that certain alterations are associated with worse responses to first-line pSACT. In particular, *ARID1A* mutations seem to be a mechanism of primary resistance to cisplatin and gemcitabine.^148^ The PARP inhibitor olaparib acts by blocking alternative DNA damage repair pathways used by cancer cells with HRD, leading to cell death (synthetic lethality). Current ESMO guidelines recommend olaparib for patients with *BRCA1/2* or *PALB2* mutations,^23^ but neither the FDA nor EMA has approved it for this indication. A phase II trial assessing olaparib in patients with pretreated advanced BTC demonstrated a PFS rate at 8 weeks of 73% with a median PFS of 16 weeks; ORR was 6.5% with a DCR of 74%. A CR and a PR were observed in patients with an *ATM* mutation and a *BRCA1* mutation, respectively.^142^

### RAS mutations

While *KRAS* G12C mutations are rare, other *KRAS* variants have a high prevalence, ranging from 10% in intrahepatic CCA and GBC to >30% in perihilar and distal CCA.^150^ Apart from other *KRAS* G12 mutations, with the G12D being the most common one, other frequent alterations include the Q61H and the A146T.^150^ Zoldonrasib is an oral *KRAS* G12D inhibitor that has demonstrated an overall good safety profile, with nausea (27%) and diarrhea (20%) being the most common TRAEs at the recommended phase 2 dose. Data in BTC are underway, but it has shown promising efficacy in pancreatic cancer, with a DCR of 80%, an ORR of 30%, and a complete clearance of the mutation on liquid biopsy during treatment in 39% of the cases.^151^ Daraxonrasib is an oral pan-RAS inhibitor directed against the active form of wild-type and mutant RAS isoforms (NRAS, KRAS, HRAS),^152^ that is currently under investigation in a phase I/Ib trial (NCT05379985).

### Other alterations

*Mouse double minute 2 homolog* (*MDM2*) amplifications are encountered in 5%–10% of patients with BTC, primarily in those with intrahepatic CCA or GBC.^90,153,154^ MDM2 is an oncoprotein that promotes p53 degradation, thus increasing tumor growth. Brigimadlin is an oral drug that interferes with the MDM2–p53 interaction, restoring p53 activity in tumor cells with wild-type *TP53*. In a phase Ia/Ib trial, among 16 patients with BTC, single-agent brigimadlin or in combination with immunotherapy led to disease control in >80% of the patients and was well tolerated, with nausea being the most common AE.^143^ A phase IIa/IIb basket study is ongoing.^144^

Deletion of *MTAP* occurs in 15% of BTC.^155,156^ *CDKN2A* is a neighboring gene on chromosome 9p21, and co-deletion is frequently observed: in a case series including >2500 intrahepatic CCAs, 16% showed *MTAP* loss and of these 98.7% had *CDKN2A* and 9p21 co-deletion.^157^ PRMT5 is a synthetic lethal target in tumors with *MTAP* homozygous deletion. Preliminary phase I data showed that AMG 193, a first-in-class dual PRMT5-MTAP inhibitor, leads to an ORR of 21.4% and a DCR of 54.8%; responses were durable and reflected a clearance of ctDNA. Four patients with BTC were enrolled in the efficacy cohort, and 3 of them achieved disease control.^56^

Claudin 18.2 is a transmembrane protein found in tight junctions. Positivity for claudin 18.2 (defined as moderate or strong expression in >75% of the cells) can be assessed via IHC and is identified in 5% of BTC, with rates up to 15% in GBC.^158^ Sonesitatug vedotin (AZD0901) is an antibody-drug conjugate targeting claudin 18.2 that showed preliminary efficacy in patients with advanced gastric cancer.^159^ The phase II CLARITY-PanTumor01 trial is an open-label basket study, including a sub-cohort for BTC, set out to determine the efficacy of single-agent treatment in this subgroup of patients.^145^ In addition, the phase I/II TWINPEAK basket study is assessing spevatamig, a bispecific antibody directed against claudin 18.2 and CD47, a membrane protein involved in tumor microenvironment regulation and phagocytosis evasion (NCT05482893).

Lastly, *TP53* mutations are the most common ones in BTC, being identified in up to 40% of the cases;^107^ in a large case series of >1600 patients, the most frequent one was the Y220C.^107^ It is also the only actionable variant. A compound (rezatapopt) is under investigation in a phase I/II basket trial. It has demonstrated good tolerability, with 79% TRAEs, but mostly grade 1 or 2, and no dose-limiting toxicities. Preliminary data on disease control indicate an ORR of 24%, but the complete results are underway.^146^

### Cell-based therapies

Cell-based therapies, such as chimeric antigen receptor-T cell (CART) and CAR-macrophage (CAR-M) therapy, have been assessed in early phase trials. In a cohort of 11 patients with HER2-positive BTC (>50% cells expressing HER2), treatment with CART-HER2 cells yielded a DCR of 55% (1 PR and 5 SD), with a median PFS of 4.8 months; no cases of severe cytokine release syndrome (CRS) were observed, and all TRAEs were reversible.^160^ CT-0508 is an anti-HER2 CAR-M, which was assessed in 14 patients with HER2 3+ or 2+/ISH-amplified tumors, including 1 with HER2 3+ BTC, who achieved an SD; treatment was overall well-tolerated, but clinical efficacy was short-lived (median PFS: 1.47 mo) and positively correlated with HER2 3+ status.^161^ In another study, 19 patients with EGFR-positive (>50%) BTC were treated with CART-EGFR cells, with similar results (1 CR and 10 SD; median PFS: 4 mo), but a higher rate of grade 3 acute fever.^162^ Moreover, in a trial of claudin 18.2-directed CART therapy for patients with gastrointestinal malignancies, 2 patients with BTC were included: one had a PR while the other had PD as best response; across all tumor types, DCR was 73% and ORR was 49%, with CRS being observed in 95% of patients, but never above grade 2.^163^ Another strategy is represented by T cell receptor fusion constructs (TRuC), which have shown greater antitumor activity then CARTs in murine models as the former utilize the entire T cell receptor, while the latter include just one of its 6 subunits.^164^ Gavo-cel is an anti-mesothelin TRuC which was evaluated in 32 patients, including one with CCA, who experienced a PR, albeit short-lived; at the recommended phase 2 dose, no grade 5 TRAEs were observed, but 61% of patients developed grades 3–4 CRS.^165^ Overall, this area of research is still in its initial phases in BTC, and all the data are preliminary.

## CONCLUSIONS

Targeted therapies have changed the treatment landscape of BTC. Despite yielding a tangible improvement in clinical outcomes, they represent a treatment option for a minority of patients with actionable targets who remain fit upon PD to first-line pSACT. There are still knowledge gaps to be addressed: from the most appropriate collocation in the treatment algorithm to the identification of patients who are primary refractory to targeted therapies, as well as how to overcome acquired resistance. While current research is set out to elucidate these issues, clinical trials investigating new drugs directed against novel targets are underway, painting the picture of an expanding and progressively more intricate field.
